# Digital therapeutics into geriatric cardiovascular emergency care

**DOI:** 10.3389/fdgth.2026.1673080

**Published:** 2026-02-10

**Authors:** Xing Hu, Zhimin Wei, Meilin Liu, Hui Geng, Haifeng Zhang

**Affiliations:** 1 Health Service Department of the Guard Bureau of the Joint Staff Department, Beijing, China; 2 Department of Geriatrics, Peking University First Hospital, Beijing, China

**Keywords:** aged, artificial intelligence, cardiovascular emergency care, digital therapeutics, the old adults

## Abstract

This mini review investigates the applications of digital therapeutics (DTx) and artificial intelligence (AI) in geriatric cardiovascular emergency care. Key elements include AI-driven biosensing for real-time risk stratification, blockchain-based secure data interoperability, tele-rehabilitation frameworks, and emerging technologies such as digital twins and brain-computer interfaces. Clinical validations shows that AI-enhanced portable ultrasound systems integrated with virtual reality (VR) optimizes diagnostic protocols and resuscitation workflows, while machine learning models achieve superior accuracy in predicting readmission risks and improving medication adherence. Notable research advances included: (1) Compared with conventional monitoring, AI biosensing improved arrhythmia detection sensitivity; (2) Deep learning models were superior to traditional methods in predicting cardiovascular events; (3) VR-assisted cardiac rehabilitation reduced anxiety scores; (4) The predictive readmission algorithm achieved high accuracy through frailty-comorbidity integration; (5) chatbot guided intervention improved medication adherence. However, limitations remain in this field, particularly in addressing age-related data biases and ethical challenges surrounding algorithmic transparency. Future researches should prioritize developing adaptive interfaces for elderly users, and advancing biocybernetic human-machine interfaces capable of stabilizing autonomic dysregulation. Importantly, these innovations must be validated in conjunction with geriatrics to ensure equitable implementation across diverse older populations.

## 1 Introduction

Cardiovascular Diseases (CVDs), including coronary artery disease and heart failure is demonstrated to be more prevalent due to the significant increase of global population aged over 60 years, reaching 1.4 billion by 2030 and 2.1 billion by 2050 (1). Such prevalence combined with CVDs' 24.3% contribution of total mortality among adults aging over 70 years (2), underscores the urgent need for targeted interventions.

Despite recent progress in cardiovascular therapeutics, the development of prehospital emergency care systems remains critically constrained. Currently, emergency medical services (EMS) is only utilized in 16.5% of potential CVD patients, of which the frequent delay still causes suboptimal clinical outcomes (3). These gaps in prehospital care highlight the urgent need for solutions integrated with novel techniques to improve emergency response and management for this aged population.

Digital therapeutics (DTx) coupled with artificial intelligence (AI) and ultra-reliable networks were demonstrated to accelerate analyses and decision-making, which serve as promising techniques to support EMS (4, 5).

In this review, we comprehensively summarized how the applications of DTx could help in acute CVD management, aiming to demonstrate the current situation, limitations and further therapeutic potential of utilizing DTx in the field of CVD. We adopted a narrative review framework for literature collation. The searched databases included PubMed, Scopus,Web of Science and CNKI. The keyword combination was: (geriatric OR elderly) AND (cardiovascular emergency OR acute cardiac event) AND (artificial intelligence OR AI OR digital therapeutics OR DTx). Most of the retrieved literatures were published between January 2018 and March 2025, with some content extending the search to 2013.

Inclusion criteria: a. Study population aged ≥65 years; b. Focus on cardiovascular emergency scenarios; c. Involving the application of AI/DTx technologies (including biosensing, diagnosis, rehabilitation, data security, etc.); d. English original studies, Meta-analyses, and high-quality reviews. Exclusion criteria: a. Basic experimental studies without clinical application orientation; b. Duplicate publications, conference abstracts, and low-quality literatures. Finally, 44 literatures were included after independent screening and cross-validation by two researchers, serving as the core evidence for this review. There is a possibility of selection bias in this review, as its primary purpose is to summarize new methods and potential technologies for geriatric cardiovascular diseases rather than to comprehensively compile all existing evidence. The first part of the review introduces the technical foundation, including the biosensing system and data security based on blockchain technology. The second part presents the applications of digital therapeutics in the emergency rescue chain, including: 1. daily heart health assessment and risk stratification system; 2. portable ultrasound during pre-hospital emergency care, and the intelligent bystander CPR guidance system; 3.medication adherence chatbot and readmission risk stratification system after hospital discharge. The third part lists the limitations of DTx and summarizes several challenges faced in future exploration.

## 2 Technological foundations

### 2.1 Advanced biosensing systems

The remote cardiac monitoring system optimizes cardiovascular care for the elderly through a non-invasive, real-time biosensing platform. Wearable patches and smart devices enable continuous electrocardiogram (ECG) monitoring, which is more convenient and has a longer duration than traditional Holter monitoring. A single-lead portable electrocardiograph device, has an increase of 3.0% (95% CI: 1.8%–4.1%) in atrial fibrillation detection rate than traditional Holter during long-term monitoring (≤14 days) (6). Similarly, another patch provides real-time arrhythmia monitoring and analysis, with a significantly higher diagnostic rate (7). Smartwatches equipped with ECG functionality perform excellently in atrial fibrillation detection, with a sensitivity of approximately 87%–92% and a specificity ranging from 95% to 99% (8). Minggang Shao et al. developed an Android APP that displayed ECG waveforms in real time and transmitted ECG data every 30s to a remote cloud server, which was analyzed and pushed to the doctor (9). By merging longitudinal physiological data with clinical analytics, such biosensing systems could detect early signs of acute coronary syndrome and decompensated heart failure, thus enabling early warning. However, there are some parts of the sensing system that are worth improving. For example, first, the sensor signal noise increases in the moving state. Second, the detection sensitivity of single-lead ECG is lower than that of 12-lead ECG.

### 2.2 Data security sharing based on blockchain technology

AI and blockchain technology show great potential in improving the efficiency and safety of emergency cardiovascular care for the elderly (10). Traditional emergency care systems often face challenges such as data silos and privacy leaks. Blockchain-mediated consent management framework for cross-institutional data sharing enables undelayed integration of patient information across different healthcare settings, including pre-hospital and in-hospital environments (11). Blockchain technology provides a decentralized and secure protection framework for recording patients’ informed consent and data access rights. Through encryption technologies such as smart contracts, the framework ensures that data sharing is both secure and efficient (12). The integration of AI with blockchain further optimizes the emergency care process. AI algorithms can analyze real-time data from wearable devices and electronic health records, predict potential cardiovascular events, and support clinical decision-making. It should be noted that when the number of nodes in the blockchain increases, the data synchronization delay may be prolonged, affecting the real-time feedback in the first aid scenario. In addition, the existing data set may have age bias (the proportion of elderly patients is insufficient), resulting in the prediction accuracy of cardiovascular events in the elderly population is lower than that in the young population.

## 3 Emergency application

### 3.1 Prehospital evaluation and risk stratification

#### 3.1.1 AI-driven gait analysis for pre-hospital assessment

The integration of AI with gait analysis has been shown to be particularly valuable in identifying early signs of functional decline in elderly patients. A multi-sensor wearable system (INDIP) has been developed for assessing gait in real-world conditions, demonstrating excellent validity and feasibility across patient cohorts, including those with heart failure (HF). This system integrates complementary sensing approaches, including plantar pressure insoles, inertial measurement units, and distance sensors, to provide comprehensive gait analysis (13).

Smartphone-based applications have also emerged as practical tools for gait assessment in older adults. Studies have demonstrated that smartphones can reliably measure spatiotemporal gait parameters such as gait speed and stride length, which are critical indicators of functional status and predictors of adverse outcomes (14). These mobile applications offer the advantage of wide application and low cost, making them highly suitable for continuous monitoring and early detection of gait abnormalities in the elderly.

Moreover, machine learning techniques have been successfully applied to predict clinical frailty scales in elderly HF patients using gait analysis. A study by Mizuguchi et al. (2024) developed a machine learning-based automatic rating system (light gradient boosting machine, LightGBM) model for the clinical frailty scale (CFS) in elderly HF patients (15). This system utilized gait parameters recorded by the smartphone camera, which are highly consistent with the evaluation results of cardiologists. The predicted CFS is also independently associated with the risk of all-cause mortality, highlighting its potential in prehospital assessment. Meanwhile, to address issues of digital literacy and sensory impairments, digital therapeutic tools have adopted personalized design features and deployment strategies. AI-powered chatbots provide elderly users with voice-guided navigation, simplified interfaces, and real-time assistance to compensate for cognitive decline and visual/hearing impairments. Elderly populations in rural areas face a wider digital divide due to factors such as limited internet access, financial constraints, and lower educational attainment. Incorporating face-to-face training delivered by community health workers and family members could be considered to improve utilization rates. These personalized approaches enhance the applicability of digital therapeutics across diverse elderly subgroups.

#### 3.1.2 AI-driven risk stratification

By leveraging AI-driven models, such as deep learning (DL) and neural multi-task logistic regression (NMTLR), real-time analysis of multi-source data, including electronic health records, biomarker trends, and wearable device outputs, enables precise risk assessment and timely intervention (16, 17). These models perform conventional methods, demonstrating enhanced predictive accuracy (e.g., C-index = 0.662 for DL models vs. 0.634 for Cox models), particularly in identifying high-risk older adults through continuous physiological monitoring (16). For instance, AI systems can detect subtle anomalies in blood pressure, glucose levels, and cardiac rhythms, facilitating early stratification of individuals at risk of acute cardiovascular events (18). Furthermore, unsupervised machine learning techniques, such as clustering analysis, were reported to be able to improve risk categorization by integrating heterogeneous data (16, 17). However, ethical concerns, including data privacy and algorithmic bias, necessitate ongoing validation in diverse elderly cohorts (17, 18). It is noteworthy that frailty and multimorbidity in elderly patients may lead to heterogeneity in their physiological signals, affecting the consistency of AI risk stratification results. In the future, further integration of geriatric-specific feature models could be pursued to better align with the physiological characteristics of the target population and enhance stratification accuracy.

### 3.2 AI-enhanced pre-hospital emergency support

#### 3.2.1 Diagnostics in pre-hospital emergency

The convergence of AI with portable ultrasound systems is revolutionizing diagnostic accuracy in pre-hospital diagnosis, especially among the elderly population. For instance, the diagnosis of chest pain represent one of the most frequent clinical scenarios in pre-hospital emergency care, encompassing conditions such as coronary heart disease, pericardial effusion, pneumothorax, aortic dissection, etc. Contemporary advances demonstrate that convolutional neural networks (CNNs) achieve 80% diagnostic accuracy for pneumothorax detection using limited training datasets (*n* = 30) (19). Hernandez-Torres et al. (2024) assessed different AI model architectures for point-of-care ultrasound diagnostics, demonstrating that models like MobileNetV2 and DarkNet53 can achieve over 85% accuracy in M-mode scans for detecting conditions such as pneumothorax and hemothorax (20). AI-driven ultrasound analysis can provide real-time guidance, reducing the skill threshold required for accurate image acquisition and interpretation. But real-world deployment is constrained by device portability and operator training. This integration has the potential to make decision-making and intervention more efficient, thereby enhancing the standardized treatment outcomes in cases such as those involving elderly patients who require prompt treatment.

#### 3.2.2 Bystander cardiopulmonary resuscitation support systems

In the scenario of out-of-hospital bystander cardiopulmonary resuscitation (CPR), AI-driven voice interaction systems, such as GPT-4o, can provide real-time, step-by-step guidance to untrained individuals. This technology could significantly improving survival outcomes (21). Similarly, AI-enhanced telemonitoring systems integrated with mobile ultrasound and 5G networks enable real-time risk assessment and decision-making, supporting timely treatment in cases of severe trauma and cardiac emergencies. For example, systems like Cobot PROMETHEUS III combine AI algorithms with cloud computing to provide immediate severity assessment and remote diagnosis (22). Such AI-driven voice guidance systems have demonstrated effectiveness in simulated environments, but their validation in real-world emergency scenarios—such as chaotic prehospital settings and elderly patients with atypical symptoms—remains unclear. Prospective trials are needed to confirm whether the survival benefits observed in simulations can be translated into actual clinical outcomes.

### 3.3 Rehabilitation management

#### 3.3.1 AI-driven medication adherence

AI-driven digital therapeutics enhance medication adherence. Systematic analysis of 12 trials (participant range: 24–412; intervention duration: 1–12 months) identified that app-based interventions prioritized medication adherence (23). And sustained improvement in adherence is beneficial for cardiovascular outcomes. Moreover, real-time adherence tracking through AI chatbots and interactive medication diaries enables dynamic adjustment of therapeutic regimens, accounting for age-related factors like cognitive decline and polypharmacy. It avoids adverse drug interactions in elderly patients taking multiple medications. The SMART-MEDS trial showed that using interactive storytelling and chatbot guided therapy engagement improved medication adherence and reduced treatment errors in an older population (24).

#### 3.3.2 Cardiac rehabilitation

VR-based cardiac rehabilitation (CR) programs have demonstrated promising results in improving patient adherence and functional outcomes. A systematic review by Garcia-Bravo et al. (2021) highlighted the potential of VR and video games as complementary tools in CR, increasing motivation and adherence among patients (25). Similarly, Shahab et al. (2022) conducted a meta-analysis showing that VR-assisted CR significantly reduced anxiety compared to standard CR (26). These findings suggest that immersive technologies can create engaging rehabilitation experiences, potentially improving long-term outcomes. Reduced visual or auditory sensitivity in elderly individuals may limit their engagement with VR-based rehabilitation programs. Adaptive design adjustments, such as adjustable sensory feedback modes and simplified interaction logic, can enhance usability and adherence. These technologies not only improve adherence and psychological outcomes but also offer innovative solutions for remote monitoring and personalized care. In real-world home-based use, factors such as elderly users’ independent operation capabilities and environmental interference may affect outcomes, requiring prospective trials for further improvement.

#### 3.3.3 Predictive readmission risk

AI-powered predictive models have demonstrated remarkable accuracy in forecasting hospital readmissions among elderly patients with cardiovascular conditions. Mohanty et al. (2022) developed ML models incorporating frailty, comorbidity, high-risk medications, and demographics, achieving a 0.79 mean area under the curve (AUC) for 30-day readmission prediction (27). Similarly, Sabouri et al. (2023) utilized ML to predict 30-day readmission and 90-day mortality in heart failure patients, with the RFE-LR model showing the best performance for in-hospital mortality (AUC: 0.91, accuracy: 0.84, specificity: 0.84, sensitivity: 0.83) (28). Li et al. (2022) focused on postoperative elderly patients, using ML to predict 30-day unplanned readmission, achieving an AUC of 0.8654 with features like operation duration and white blood cell count (29). Najafi-Vosough et al. (2021) compared six ML methods for predicting hospital readmission in Iranian HF patients, finding random forest (RF) performed best with accuracy of 0.90–0.91. These models not only improve prediction accuracy but also provide insights into key risk factors, facilitating personalized intervention strategies (30).

## 4 Research frontiers

### 4.1 Human-machine interaction

Recent advances in neuroprosthetic interfaces and implantable biosensors demonstrate transformative potential for geriatric care integration. Electroencephalography-based brain-computer interfaces (BCIs) have achieved 86.3% offline classification accuracy in controlling robotic gait orthoses through kinesthetic motor imagery, enabling spinal cord injury patients to restore ambulation with 0.812 ± 0.048 (*p*-value < 10^−4^) cross-correlation to control cues (31). Electroencephalography-based BCIs have shown promise in controlling robotic gait orthoses for patients with spinal cord injury, and we propose their theoretical potential for cardiac rhythm modulation. However, further research specifically targeting cardiovascular diseases is required to validate this potential. In addition, stent-integrated wireless pressure monitors now achieve 0.5 mmHg resolution via a microelectromechanical systems capacitive sensing (32). Machine learning algorithms analyzing stent-derived hemodynamic waveforms may predict acute coronary events pre-onset through microvascular resistance anomalies.

### 4.2 Digital twin technology

By synergizing inductive statistical models with deductive mechanism-based hemodynamic simulations (33), high-fidelity physiologically driven models may be possible. For instance, principal component analysis (PCA)-optimized ventricular motion analytics improve post-myocardial infarction exercise prescription accuracy (33). Machine learning-enhanced digital twin technology enables personalized fentanyl dosing via virtual population simulation, reducing drug-induced adverse events by 16% (34). Digital twin technology has also been effective in medical education. Notably, immersive digital twins facilitate clinical competency building, which the medical staff's medication and rescue can be truly reflected in the simulated human vital signs (35). Digital twin technology in cardiovascular care is still in the feasibility stage, with most applications limited to simulation and medical education. Its translation to real-time emergency care for the elderly requires ongoing validation and optimization in clinical trials.

### 4.3 Virtual reality in geriatric CPR training

The cross-sectional survey showed that 72.8% of resuscitation professionals supported VR as a method of simulation training (36). Randomized trials demonstrate non-inferiority in compression rate acquisition (114 ± 12 vs. 109 ± 12 compressions/min) compared to traditional methods, VR cohorts exhibit suboptimal depth control (49 ± 10 mm vs. 57 ± 5 mm, *p* < 0.001) (37). Current systems show measurement accuracy (chest compression rate 1.4/minute and chest compression depth 3.7 mm variance) yet lack adaptive biomechanical feedback for age-specific thoracic compliance (38).

**Figure 1:**
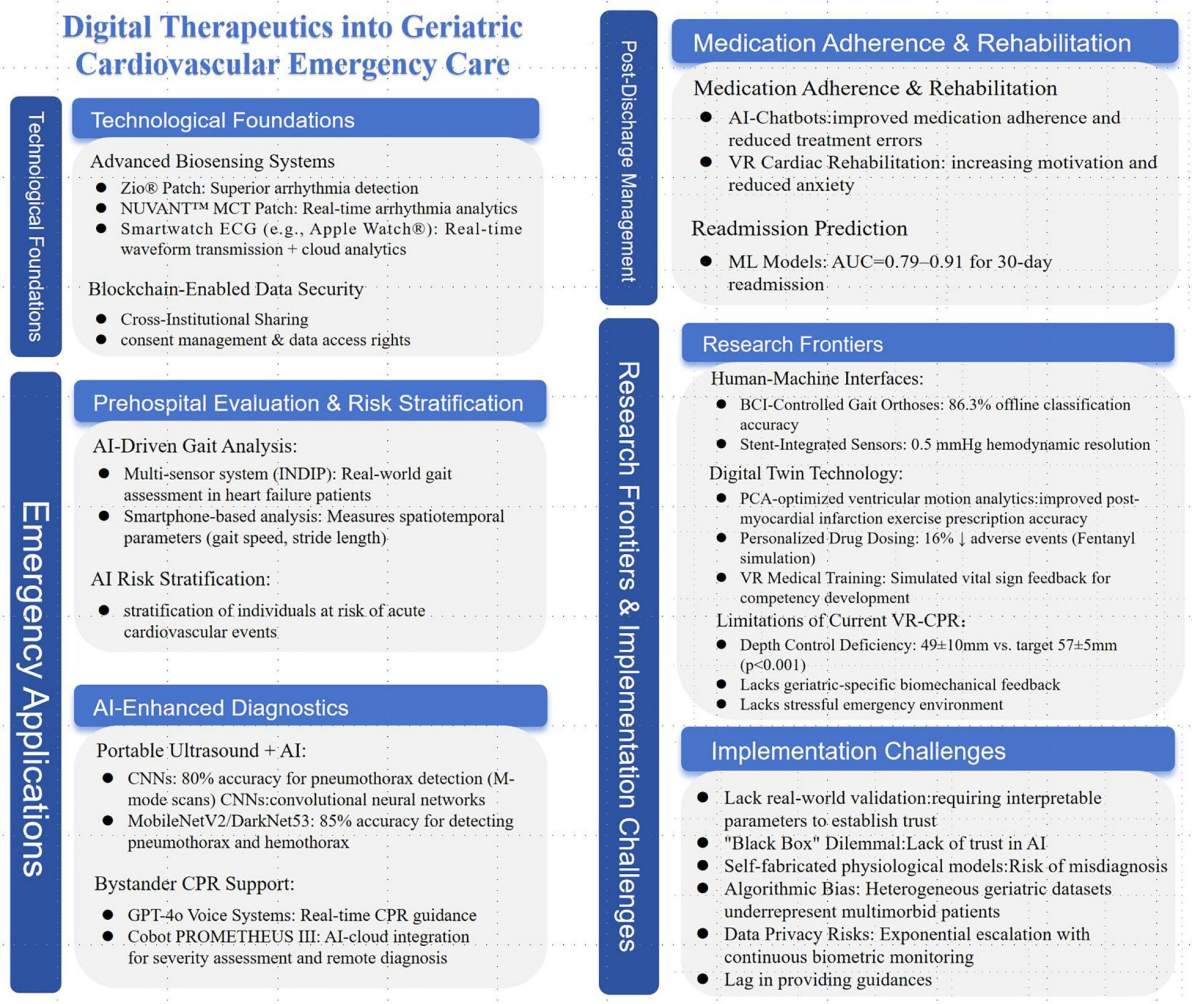
Framework of digital therapeutics into geriatric cardiovascular emergency care.

Above brief content framework can be referred to as Figure 1.

## 5 Implementation challenges and ethical-legal framework

Despite AI's proven superiority in pattern recognition for risk stratification, translational gaps persist: 88% of medical AI systems remain at the prototype stage, with only 7% achieving real-world clinical validation (39). Clinicians demand AI models to outperform human capabilities while simultaneously requiring interpretable parameters to establish trust, creating a “black box” dilemma that hinders adoption (40). Some medical artificial intelligence models inadvertently incorporate self-fabricated physiological models in the process of data enhancement, which has the risk of misdiagnosis. Distinguishing developer training errors from inherent stochastic system noise is both legally ambiguous and ethically risky (41, 42). To address implementation barriers, we recommend aligning the development of AI-driven digital therapeutics (AI-DTx) with the FDA's Software as a Medical Device (SaMD) guidelines. This will enhance the rationality and safety of the approval process, thereby promoting the development of the field through standardized procedures. For the elderly, heterogeneous geriatric datasets often lack representation of multimorbid patients, perpetuating algorithmic biases that disproportionately affect elderly populations (43). To mitigate age-related data bias, researchers should adopt targeted approaches such as data reweighting and domain adaptation–ensuring sufficient representation of frail, multimorbid elderly patients in training datasets. Meanwhile, fairness auditing can be integrated with data reweighting and domain adaptation. For instance, audit the AI model's risk prediction outcomes across different elderly subgroups to identify and rectify data biases. Even if the information provided by the AI is accurate, current AI models frequently lag in providing accurate, real-time recommendations during cardiopulmonary arrest events. At the same time, user privacy is also worthy of attention, privacy risks escalate exponentially as AI-driven continuous monitoring generates enormous biometric data, exposing people a greater risk of privacy leakage (42, 44). Blockchain-based data sharing frameworks should incorporate patient-controlled access rights via smart contracts, enabling patients to monitor access and citation times in real time. Additionally, security clauses dedicated to privacy protection should be added to further safeguard sensitive information.

## 6 Conclusion

The convergence of AI and digital therapeutics DTx transforms geriatric cardiovascular emergency care, offering tailored solutions for aging populations. AI-enhanced biosensing systems and predictive analytics demonstrate superior arrhythmia detection and risk stratification compared to conventional methods, enabling timely interventions through continuous physiological monitoring. Blockchain-powered secure data-sharing platforms address key interoperability challenges, facilitating coordinated care across pre-hospital and hospital settings. Innovations such as AI-driven portable ultrasound and virtual reality resuscitation training further optimize emergency workflows.

Machine learning models deliver high accuracy in predicting readmissions and enhancing medication adherence. Dataset biases, and ethical concerns—particularly around algorithmic transparency and data sovereignty—underscore the need for geriatric-specific validation frameworks. Future advancements must prioritize adaptive interfaces for elderly users, integrating biocybernetic technologies with patient-centric design principles. Multidisciplinary collaboration among clinicians, engineers, and policymakers is essential to navigate regulatory complexities and ensure equitable implementation. Ultimately, sustainable integration of AI-DTx ecosystems hinges on harmonizing technological innovation with ethical governance, enabling precision emergency care that meets aging populations' unique needs.
